# Complete laparoscopic removal of retropubic midurethral tape (tension-free vaginal tape) from the obturator nerve: a multidisciplinary approach

**DOI:** 10.1007/s00192-019-04016-6

**Published:** 2019-06-29

**Authors:** William R. Cooke, Rufus Cartwright, C. Anton Fries, Natalia Price

**Affiliations:** 1 Department of Gynaecology, Women’s Centre, John Radcliffe Hospital, Headley Way, Oxford, OX3 9DU UK; 2 Nuffield Department of Women’s & Reproductive Health, University of Oxford, Level 3 Women’s Centre, John Radcliffe Hospital, Headley Way, Oxford, OX3DU UK; 3 Department of Plastic Surgery, John Radcliffe Hospital, Oxford, UK

**Keywords:** Trans-vaginal tape, Complication, Obturator nerve, Obturator neuralgia, Mesh

## Introduction

Obturator neuralgia following mid-urethral sling is rare and most commonly associated with transobturator tapes [1]. Prior reports of obturator nerve injury from retropubic midurethral tape (or tension-free vaginal tape, TVT) have described leaving the portion of tape attached to the nerve in situ [2, 3]. We present a case of obturator neuralgia secondary to lateral retropubic tape misplacement. A multi-disciplinary approach resulted in complete tape removal.

## Case study

**Fig. 1:**
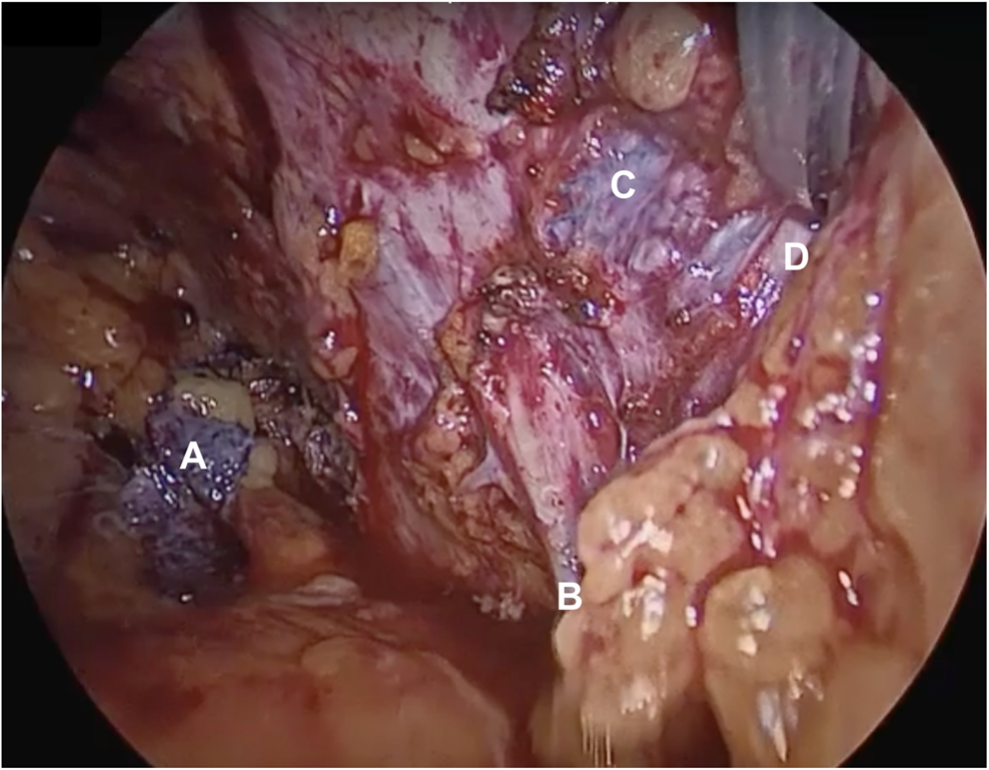
Intra-operative laparoscopic image following initial dissection. Tension-free vaginal tape (TVT) had been divided vaginally and the vaginal portion passed into the peritoneal cavity (*A*); traction on this portion demonstrated the path of the mesh. Proximity and attachment to the obturator nerve and neurovascular bundle (*B*) were noted and a plastic surgeon gave advice. The distal portion of the right TVT was adherent to the pelvic side wall (*C*) in close proximity to the external iliac vessels (*D*)

**Fig. 2:**
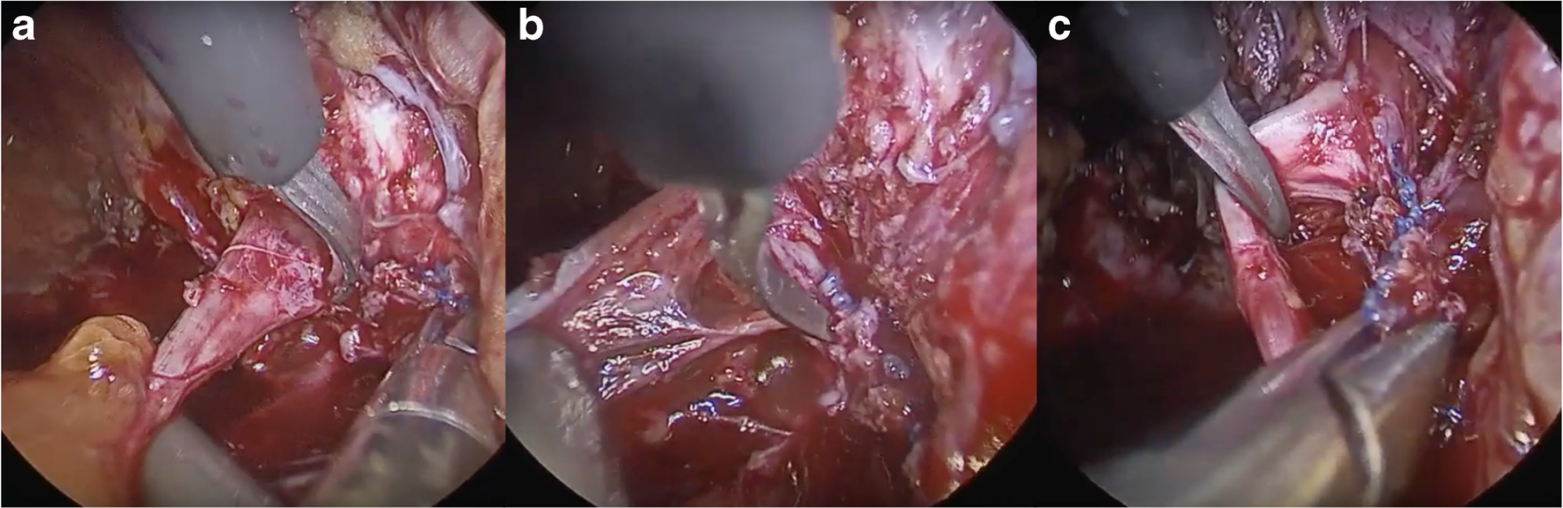
Cautious sharp and blunt dissection in close proximity to the TVT allowed the tape to be removed from the **a** posterior and **b**, **c** lateral surfaces of the obturator nerve

**Fig. 3:**
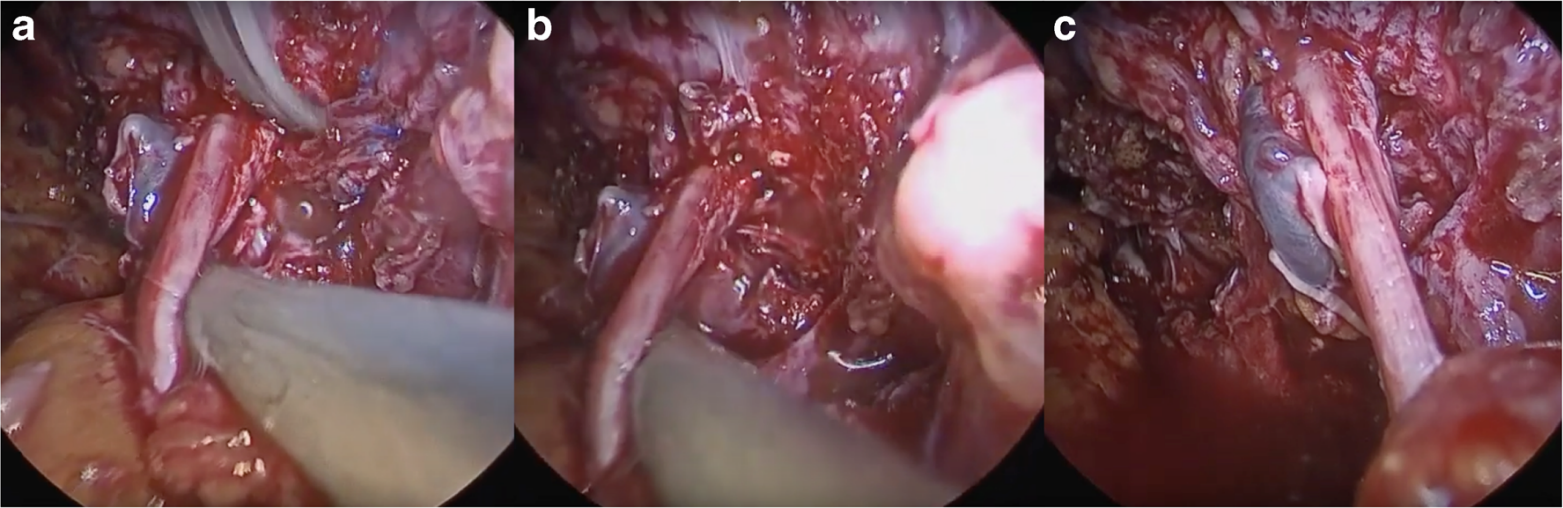
**a**, **b** Following direct dissection from the obturator nerve, the final portion of the mesh was completely removed from the internal obturator muscle and pelvic side wall. **c** Obturator nerve integrity was confirmed intra-operatively by a plastic surgeon

A 53-year-old woman had been referred to Urogynecology with right groin pain following TVT insertion 18 months earlier. She had had a previous diagnosis of fibromyalgia. She reported sharp right-sided groin pain, starting immediately on waking post-operatively. The pain radiated down her medial thigh and was exacerbated by walking. She was initially managed locally by orthopaedic and pain clinics, with neuropathic pain medications, CT-guided steroid injection to the obturator nerve and psychology input. MRI failed to show scarring in a location that might suggest tape misplacement. Following tertiary centre referral for unsuccessful conservative management of persistent pain (with no improvement in stress incontinence), her case was discussed at our multi-disciplinary team meeting and the patient offered laparoscopic TVT removal. Following dissection and division of the vaginal portion of the tape, the left intra-abdominal arm was found and removed; the right arm was not easily identified. Traction on the proximal portion of the mesh revealed that it passed through the internal obturator muscle, attached to the obturator nerve posteriorly and ended close to the external iliac vessels (Fig. 1). The mesh was carefully dissected from the epineurium of the obturator nerve (Fig. 2) with guidance from a plastic surgeon. Fascicular integrity was confirmed intra-operatively. The mesh was then completely removed (Fig. 3). At 2 months post-operatively, the patient had complete resolution of right-sided groin pain, no muscle weakness and an overall improvement in stress incontinence. The tape was an Ethicon polypropylene standard type 1 TVT mesh.

## Conclusion

During retropubic mid-urethral sling insertion, care should be taken not to advance the mesh laterally. In patients suffering from post-operative obturator neuralgia, malpositioning of the tape should be considered, although it is challenging to diagnose pre-operatively.

## Electronic supplementary material

Supplementary material Video demonstrating complete laparoscopic removal of retropubic midurethral tape (TVT) from the obturator nerve. (MP4 419059 kb)
